# Supplementary material for: GPR180 is a component of TGFβ signalling that promotes thermogenic adipocyte function and mediates the metabolic effects of the adipocyte-secreted factor CTHRC1
Source: Nat Commun. 2021 Dec 8;12:7144. doi: 10.1038/s41467-021-27442-x (PMC8655035; doi:10.1038/s41467-021-27442-x)
Supplement: Supplementary file 1 — Supplementary Information [file 41467_2021_27442_MOESM1_ESM.pdf]

Supplementary Figure 1

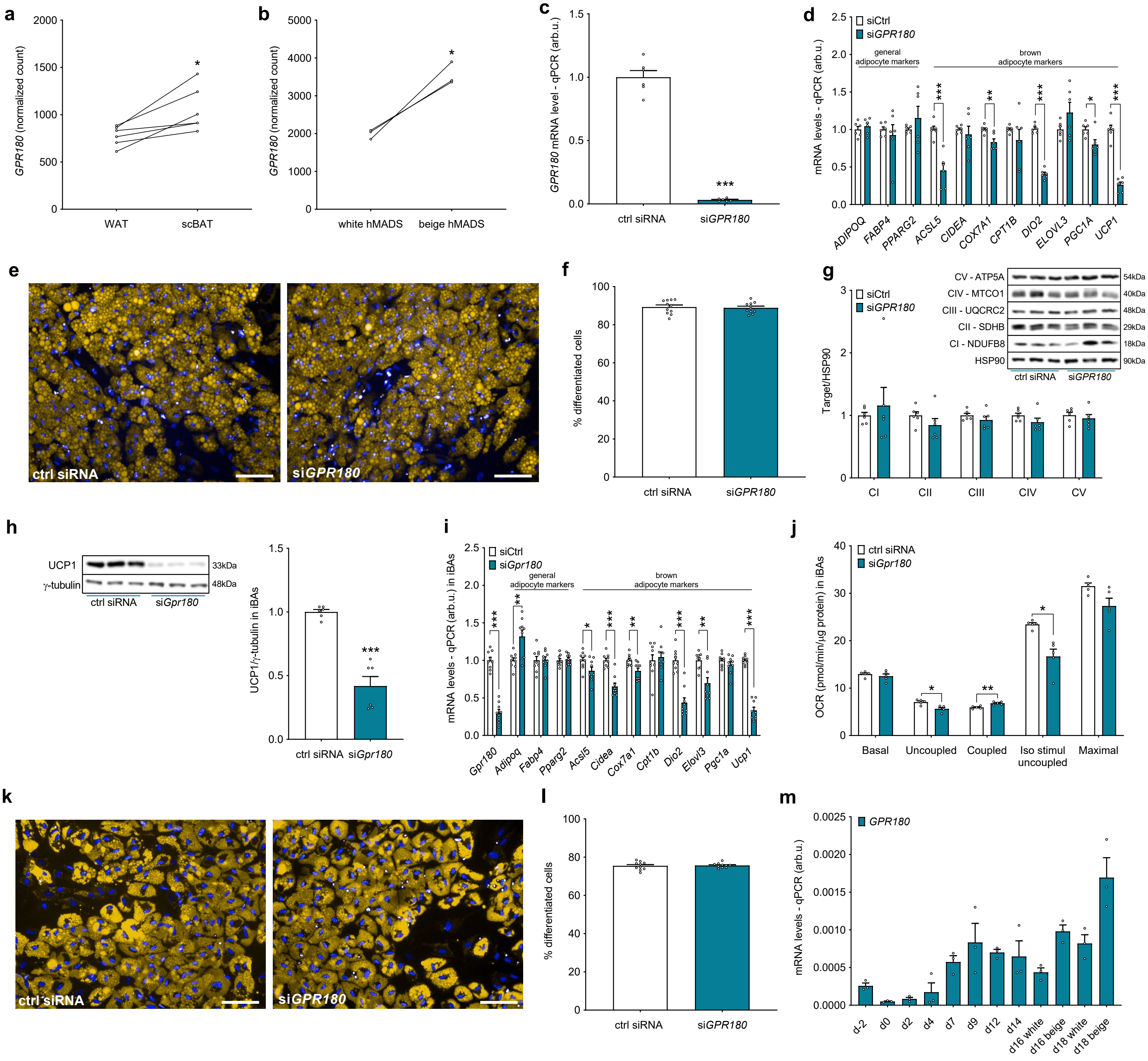

Supplementary Fig. 1 (related to Fig. 1): GPR180 is upregulated in BAT and required for brown phenotype in both human and murine adipocytes.

Expression of GPR180 in (a) human fat tissues (n=6; p=0.0167) and (b) human beige and white adipocytes (n=3;p=0.0236). (c) Knockdown efficiency of siRNAs targeting GPR180 in mature beige hMADS cells (n=6; p<0.0001). (d) Gene expression of general and brown adipocyte markers in human beige adipocytes with ablated GPR180 (n=6; *ACSL5* p=0.0003; *COX7A1* p= 0.0062; *DIO2* p<0.0001; *PGC1A* p=0.0209; *UCP1* p<0.0001). (e) Representative images with scale bar 100 µm and (f) quantification of cell differentiation following GPR180 knockdown in beige hMADS cells (n=11; p=0.7484), lipid droplets in yellow (LD540), nuclei blue (Hoechst). Experiment was repeated 3 times independently with similar results. (g) Representative blots and quantification of OXPHOS proteins in beige hMADS cells (n=6; CI p=0.5978; CII p=0.2228; CIII p=0.2977; CIV p=0.1724; CV p=0.5228). Effect of Gpr180 knockdown in murine immortalized brown adipocytes on (h) UCP1 protein (n=6; p<0.0001), (i) expression of general and brown adipocyte marker genes (n=9; *Gpr180* p<0.0001; *Adipoq* p=0.0032; *Acsl5* p=0.0389; *Cidea* p<0.0001; *Cox7a1* p=0.0071; *Dio2* p<0.0001; *Elovl3* p=0.0020; *Ucp1* p<0.0001) and (j) mitochondrial respiration (n=5; uncoupled p=0.0271; coupled p=0.0093; iso stimul uncoupled p=0.0486). (k) Representative images with scale bar 100 µm and (l) quantification of cell differentiation following GPR180 knockdown in undifferentiated preadipocytes (n=10; p=0.7917), lipid droplets in yellow (LD540), nuclei blue (Hoechst). Experiment was repeated 3 times independently with similar results. (m) GPR180 expression during time-course of hMADS cells differentiation. Data are shown as mean ± SEM. Statistical significance was calculated using paired Student´s t test (Fig. a and b), unpaired two-sided Student´s t test (Fig. c-i and l) and two-way ANOVA with Sidak post-hoc test (Fig. j) and indicated as * $p < 0.05$, ** $p < 0.01$ and *** $p < 0.001$. ACSL5, Acyl-CoA synthetase long chain family member 5; ADIPOQ, Adiponectin; ATP5A, ATP synthase F1 subunit alpha; CIDEA, Cell death inducing DFFA like effector a; COX7A1, Cytochrome c oxidase subunit 7a1; CPT1β, Carnitine palmitoyltransferase 1β; DIO2, Iodothyronine deiodinase 2; ELOVL3, Fatty acid elongase 3; FABP4, Fatty acid binding protein 4; GPR180, G protein coupled receptor 180; hMADS, human multipotent adipose derived stem cells; HSP90, Heat shock protein 90; MTCO1, Mitochondrially encoded cytochrome c oxidase1; NDUFB8, NADH:ubiquinone oxidoreductase subunit B8 PGC1A, PPARγ coactivator 1α; PPARG2, Peroxisome proliferator activated receptor gamma; scBAT, supraclavicular brown adipose tissue; SDHB, Succinate dehydrogenase complex iron sulfur subunit B; UCP1, Uncoupling protein 1; UQCRC2, Ubiquinol-cytochrome c reductase core protein 2; WAT, white adipose tissue.

Supplementary Figure 2

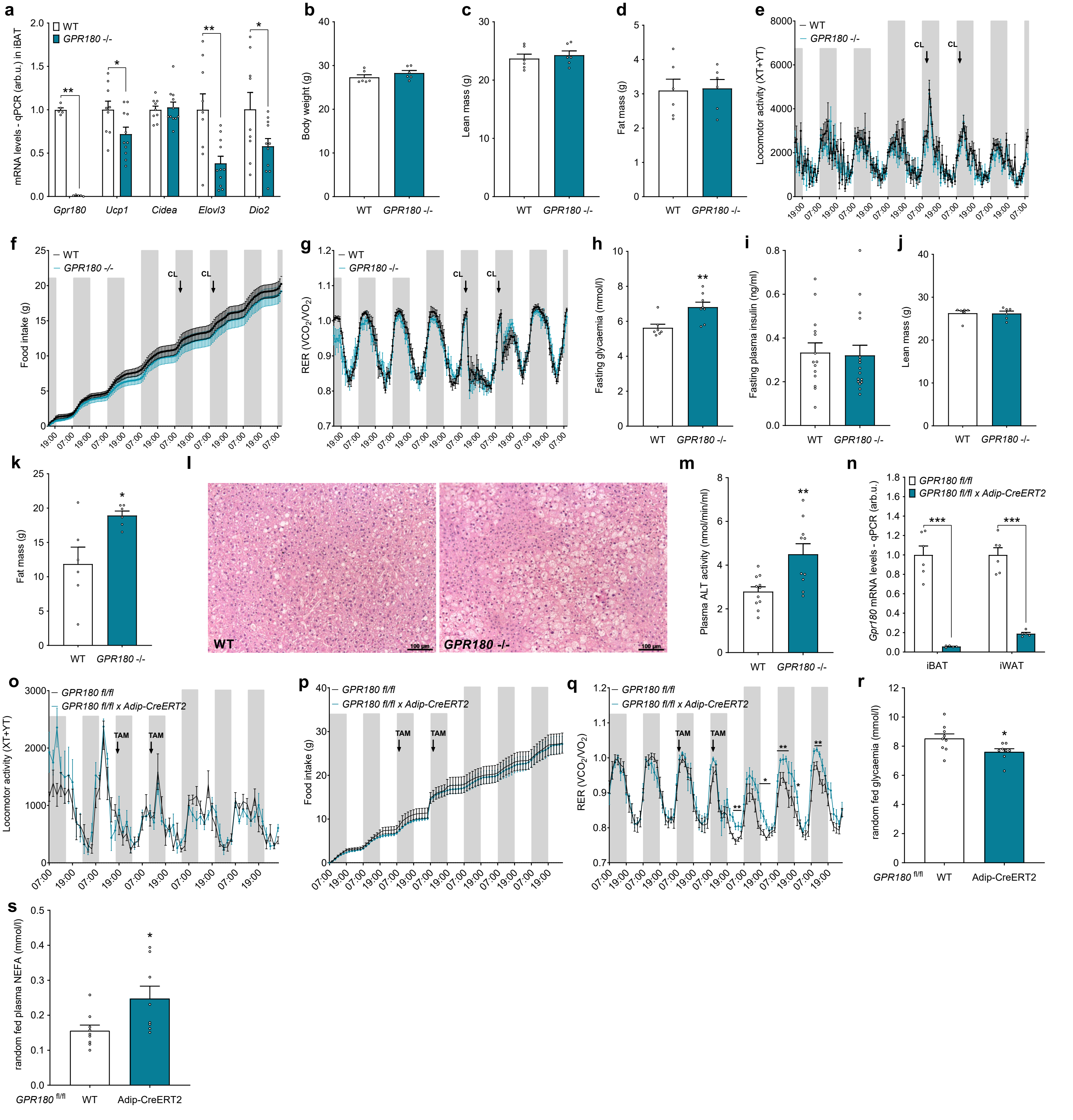

Supplementary Figure 2 (related to Fig. 2): Metabolic derangements in GPR180 knockout mice

(a) Expression of *Gpr180* (n=5; p<0.0001) and selected brown adipocyte markers in iBAT of male *GPR180* knockout mice and their wild type littermates (WT n=9 and GPR180 -/- n=11; p=0.0365 for *Ucp1*, p=0.7231 for *Cidea*, p=0.0041 for *Elovl3*, p=0.0484 for *Dio2*) housed at RT and fed chow diet. (b) Body weight (p=0.2587), (c) lean (p=0.5936) and (d) fat mass (p=0.8833) of animals housed at RT and fed chow diet (n=6). Effect of Gpr180 deletion on (e) locomotor activity (p=0.1839), (f) cumulative food intake (p=0.4613) and (g) respiratory exchange ratio (p=0.5832) (n=6). CL-316.243 (0.1 mg/kg/day) was administered intraperitoneally at indicated time points. (h) Blood glucose (WT n=7, *GPR180*-/- n=8; p=0.0059) and (i) plasma insulin level (WT n=14, *GPR180*-/- n=16; p=0.8441) in chow fed mice fasted for 6h prior to blood sampling. Body composition, (j) lean mass (p=0.9303) and (k) fat mass (p=0.0195) of mice housed at RT and challenged with HFD for 12 weeks (n=6). (l) Representative images of haematoxylin and eosin stained liver sections (10x magnification with scale bar 100 µm, experiment was repeated twice independently with similar results) and (m) plasma alanine-aminotransferase activity (p=0.0033) in global *GPR180* knockouts after 12 weeks of HFD feeding regimen at RT (WT n=11, *GPR180*-/- n=10). (n) Knockout efficiency in iBAT (p<0.0001) and iWAT (p<0.0001) of male adipocyte specific *GPR180* (a*GPR180*) knockouts and fl/fl controls (fl/fl control n=6 and a*GPR180* knockout n=5) 2 weeks after tamoxifen (TAM) gavage (2mg/animal) in two consecutive days. Effect of *GPR180* ablation in mature adipocytes on (o) locomotor activity (p=0.0538), (p) food intake (p=0.7565) and (q) respiratory exchange ratio (p=0.0132) in a*GPR180* knockouts (n=5) and fl/fl controls (n=6). Random fed (r) glycaemia (p=0.0309) and (s) plasma free fatty acids level (p=0.0265) in a*GPR180* knockouts (n=8) and fl/fl controls (n=9) on 3rd day after tamoxifen induced knockout. Data are presented as mean ± SEM. Statistical analysis was performed using two-sided Student´s t test (Fig. a-e, h-n, r and s) or two-way ANOVA with repeated measurements followed by Sidak post-hoc test (Fig. e-g, o-q). Significance is indicated as * p < 0.05, ** p < 0.01 and *** p < 0.001. ALT, Alanine aminotransferase; CIDEA, Cell death inducing DFFA like effector a; CL, CL-316,243; DIO2, Iodothyronine deiodinase 2; ELOVL3, Fatty acid elongase 3; GPR180, G protein coupled receptor 180; iBAT, interscapular brown adipose tissue; iWAT, inguinal white adipose tissue; NEFA, non-esterified fatty acids; RER, respiratory exchange ratio; RT, room temperature; TAM, tamoxifen; UCP1, Uncoupling protein 1; WT, wild type.

Supplementary Figure 3

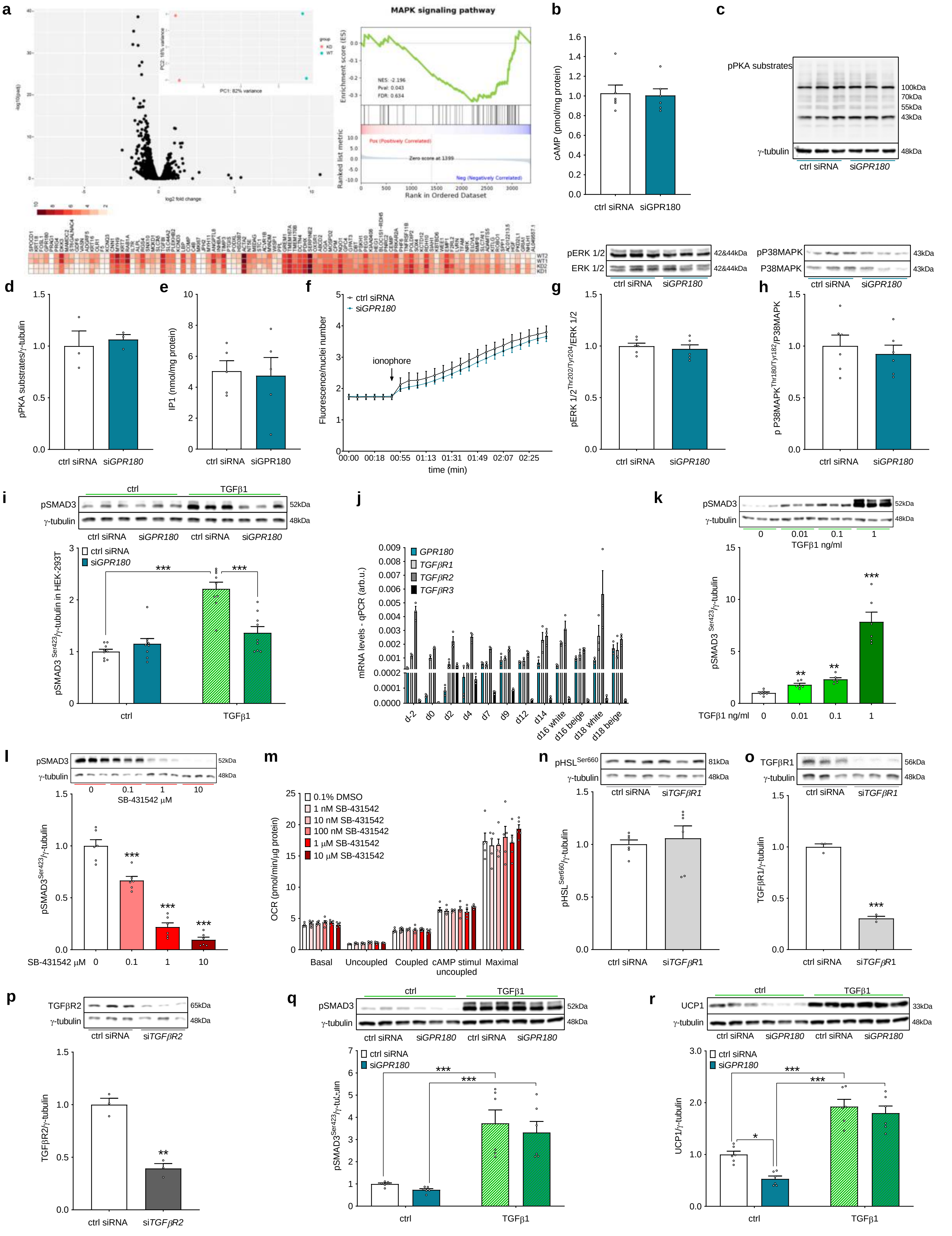

Supplementary Fig. 3 (related to Fig. 3): GPR180 is not a GPCR but a component of TGFβ signalling pathway

(a) Analysis of differentially expressed genes following GPR180 knockdown in beige hMADS cells with top 50 regulated genes shown in the bottom. DESeq2 detects DE genes based on a generalised linear model using the negative binomial distribution. The p-values obtained by Walt test were corrected by Benjamini–Hochberg multiple testing procedure. Effect of GPR180 knockdown on (b) cAMP (n=5; p=0.8448), (c, d) phospho-PKA substrates (n=3; p=0.7010) (e) IP1 (n=5;p=0.8349) and (f) calcium levels in beige hMADS cells (n=5; p=0.5480). Phosphorylation of kinases (g) ERK (at Threonine 202; p=0.5960) and (h) P38MAPK (at Threonine 180; p=0.5758) in beige hMADS cells with ablated GPR180 (n=6). (i) Effect of acute (1 hour) TGFβ1 stimulus (1ng/ml) on SMAD3 phosphorylation (at Serine 423) in HEK-293T in combination with GPR180 silencing (n=9; p<0.0001 for ctrl siRNA ctrl vs ctrl siRNA TGFβ1; p<0.0001 for ctrl siRNA TGFβ1 vs si*GPR180* TGFβ1). (j) Expression of genes encoding GPR180 and individual TGFβ receptors in time course of hMADS cells differentiation (n=3). (k) Phosphorylation of SMAD3 (at Serine 423) in response to long-term (72 hours) TGFβ1 treatment of hMADS cells (n=6); p=0.0022 for 0.01 ng/ml, p=0.001 for 0.1 ng/ml and p<0.0001 for 1ng/ml). Effect of long-term TGFβR1 pharmacological inhibition (72 hours) on (l) SMAD3 phosphorylation (at Serine 423) (n=6; p<0.0001 for all comparisons) and (m) mitochondrial respiration (n=5; p=0.5209). (n) Phosphorylated HSL (at Serine 660) in beige hMADS cells with ablated TGFβR1 (n=6; p=0.6598). Knockdown efficiency after (o) TGFβR1 (p<0.0001) and (p) TGFβR2 siRNA mediated silencing (n=3; p=0.0014). Effect of long-term (72 hours) TGFβ1 treatment (1ng/ml) on (q) phosphorylated SMAD3 (at Serine 423; p=0.0005 for TGFβ1 treatment within ctrl siRNA and p=0.0009 for TGFβ1 treatment within si*GPR180*) and (r) UCP1 protein in beige adipocytes lacking GPR180 (n=6; p=0.0316 for ctrl siRNA ctrl vs si*GPR180* ctrl; p<0.0001 for ctrl siRNA ctrl vs ctrl siRNA TGFβ1 and p<0.0001 for si*GPR180* ctrl vs si*GPR180* TGFβ1). Data are presented as mean ± SEM. Statistical analysis was performed using two-sided Student´s t test (Fig. b-e, g, h, n-p), one-way (Fig. k and l) or two-way ANOVA (Fig. f, i, m, q and r) with Dunnett's and Sidak post-hoc test, respectively. Significant differences are indicated as * p < 0.05, ** p < 0.01 and *** p < 0.001. cAMP, cyclic adenosine monophosphate; DMSO, dimethyl sulfoxide; ERK, Extracellular signal regulated kinase; GPR180, G protein coupled receptor 180; HSL, Hormone sensitive lipase; IP1, inositol monophosphate; MAPK, Mitogen-activated protein kinase; OCR, oxygen consumption rate; PKA, Proteinkinase A; SMAD3, Mothers against decapentaplegic homolog 3; TGFβ1, Transforming growth factor β1; TGFβR1, Transforming growth factor β receptor type 1; TGFβR2, Transforming growth factor β receptor type 2; TGFβR3, Transforming growth factor β receptor type 3; UCP1, Uncoupling protein 1.

Supplementary Figure 4

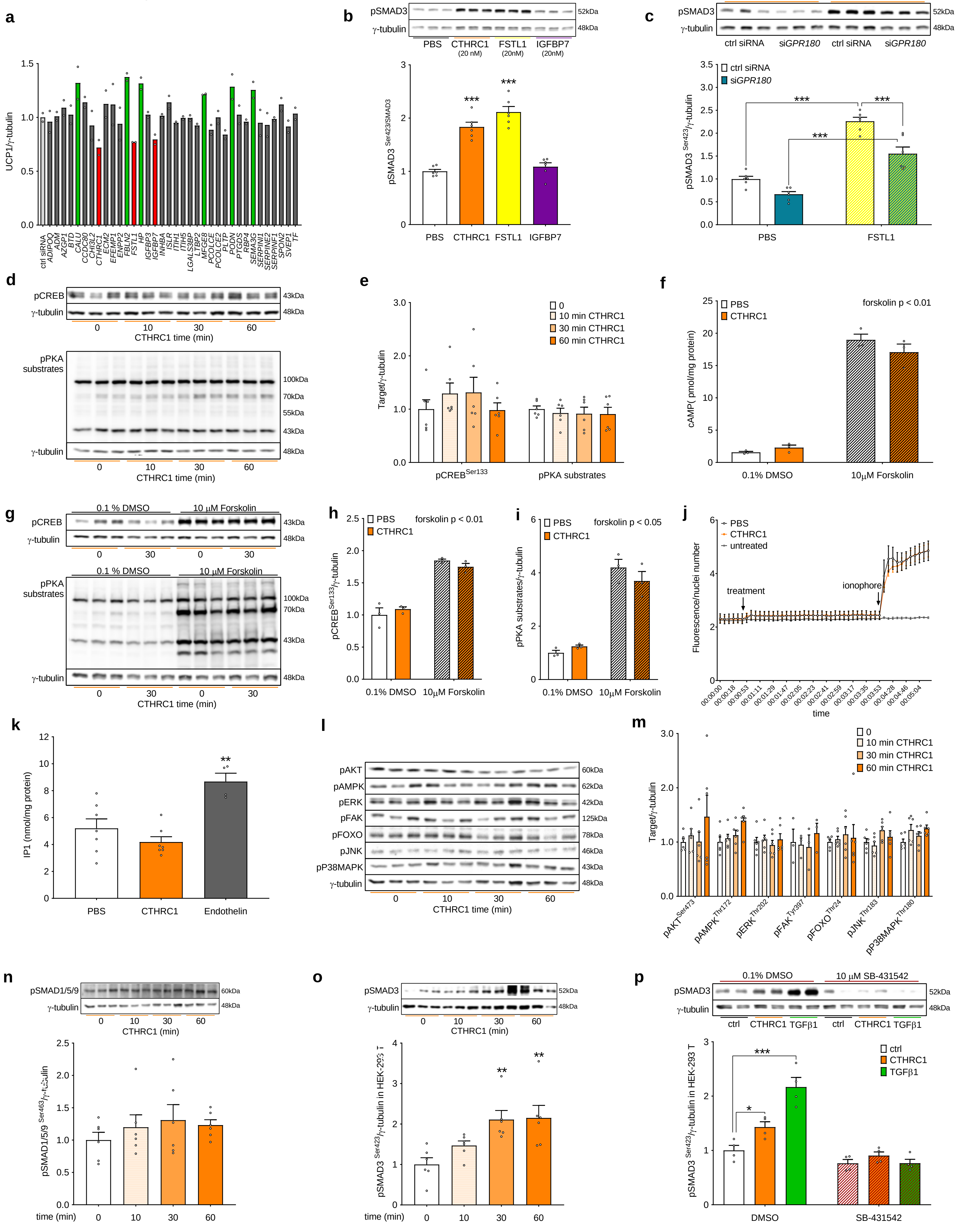

Supplementary Fig. 4 (related to Fig. 4): CTHRC1 does not activate GPCR mediated signalling

(a) Screening aimed to identify secreted molecules regulating UCP1 protein expression based on siRNA-mediated silencing of individual target genes; ≥ 20% upregulation in green, ≥ 20 % downregulation in red (n=2). (b) Representative blot and quantification of SMAD3 phosphorylation (at Serine 423) in beige hMADS cells in response to CTHRC1, FSTL1 and IGFBP7 (20nM; 1 hour) treatment (n=6; p<0.0001 for both CTHRC1 and FSTL1). (c) Representative blot and quantification of SMAD3 phosphorylation (at Serine 423) in beige hMADS cells in response to FSTL1 treatment (20nM; 1 hour) combined with *GPR180* knockdown (n=6; p<0.0001 for ctrl siRNA PBS vs ctrl siRNA FSTL1; p<0.0001 for si*GPR180* PBS vs si*GPR180* FSTL1 and p=0.0002 for ctrl siRNA FSTL1 vs si*GPR180* FSTL1). (d) Representative blots and (e) quantification of short-term CTHRC1 treatment (500ng/ml) on phosphorylation of CREB (at Serine 133; p=0.5261) and phospho-PKA substrates (p=0.9142) in human beige adipocytes starved for 2h prior the treatment (n=6). (f) Cyclic AMP levels in basal and forskolin (10μM) pre-treated cells in response to acute CTHRC1 (500ng/ml) treatment (n=3; forskolin p=0.0028, siRNA p=0.2746). (g) Representative blots and quantification of CTHRC1 action on phosphorylation of (h) CREB (at Serine 133; forskolin p=0.0100, siRNA p=0.972) and (i) phospho-PKA substrates (forskolin p=0.0130, siRNA p=0.5663) in beige adipocytes starved for 2h prior the treatment (n=3). Effect of acute CTHRC1 treatment (500ng/ml) on (j) intracellular calcium level (n=4; p=0.9453) and (k) IP1 accumulation (n=7; p=0.3558 for CTHRC1 and p=0.0035 for Endothelin). (l) Representative blots and (m) quantification of phosphorylation of various kinases in time course of CTHRC1 treatment (500ng/ml) of beige adipocytes (n=6; p=0.4427 for AKT, p=0.0522 for AMPK; p=0.8326 for ERK; p=0.8075 for FAK; p=0.9190 for FOXO1; p=0.1127 for JNK and p=0.117 for P38MAPK). (n) SMAD1/5/9 phosphorylation (n=6; p=0.6248) in hMADS cells in response to CTHRC1 (500 ng/ml). (o) Effect of CTHRC1 stimulation (500ng/ml) on SMAD3 phosphorylation (at Serine 423) in HEK-293T cells (n=6; p=0.0046 for 30 min and p=0.0034 for 60 min). (p) Effect of short-term (1 hour) CTHRC1 stimulus on SMAD3 phosphorylation (at Serine 423) in HEK-293T cells pre-treated for 2h with TGFβR1 kinase inhibitor SB-431542 (n=4; p=0.0259 for CTHRC1 in DMSO and p<0.0001 for TGFβ1 within DMSO). Data are presented as mean ± SEM. Statistical significance was calculated using one- (Fig. b, e, k, m-o) or two-way ANOVA (Fig. c, f, h-j and p) with Dunnett's and Sidak post-hoc test, respectively and is indicated as * p < 0.05, ** p < 0.01 and *** p < 0.001. AKT, AKT serine/threonine kinase 1; AMPK, Protein kinase AMP-activated catalytic subunit alpha 2; cAMP, cyclic adenosine monophosphate; CREB, cAMP responsive element binding protein 1; CTHRC1, Collagen triple helix repeat containing 1; DMSO, dimethyl sulfoxide; ERK, Extracellular signal regulated kinase; FAK, Protein tyrosine kinase 2; FOXO, Forkhead box O; FSTL1, Follistatin like 1; GPR180, G protein coupled receptor 180; IGFBP7, Insulin like growth factor binding protein 7; IP1, inositol monophosphate; JNK, c-Jun N-terminal kinase; MAPK, Mitogen-activated protein kinase; PBS, phosphate buffered saline; PKA, Proteinkinase A; SMAD3, Mothers against decapentaplegic homolog 3; TGFβ1, Transforming growth factor β1.

Supplementary Figure 5

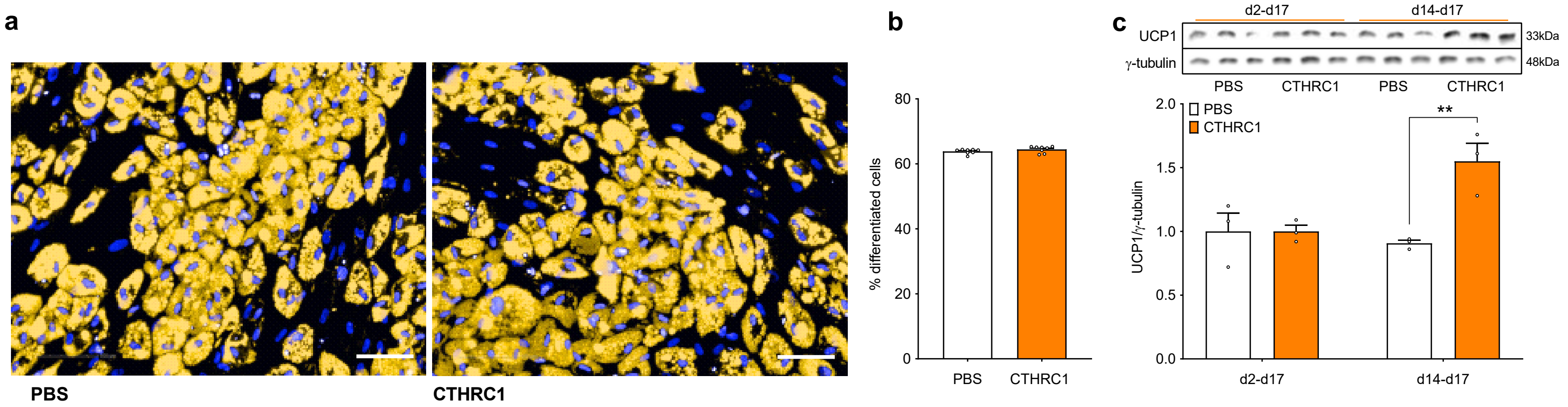

Supplementary Fig. 5 (related to Fig. 5): CTHRC1 does not affect adipocyte formation

(a) Representative images and (b) quantification of the CTHRC1 (500ng/ml) effect on adipocyte differentiation (n=8; p=0.1805); staining of lipid droplets (yellow) and nuclei (blue); scale bar 100 µm, the experiment was repeated three times independently. (c) Effect of CTHRC1 treatment (500ng/ml) on beige adipocyte formation (treatment day 2-17) and adipocyte browning (treatment day 14-17) (n=3; p=0.0147 for CTHRC1 within d14-d17). Data are shown as mean ± SEM. Statistical analysis was performed by unpaired two-sided Student´s t test (Fig. b) and two-way ANOVA with Sidak post-hoc test (Fig. c). Significance is indicated as * p < 0.05, ** p < 0.01 and *** p < 0.001. CTHRC1, Collagen triple helix repeat containing 1; PBS, phosphate buffered saline; UCP1, Uncoupling protein 1.

Supplementary Figure 6

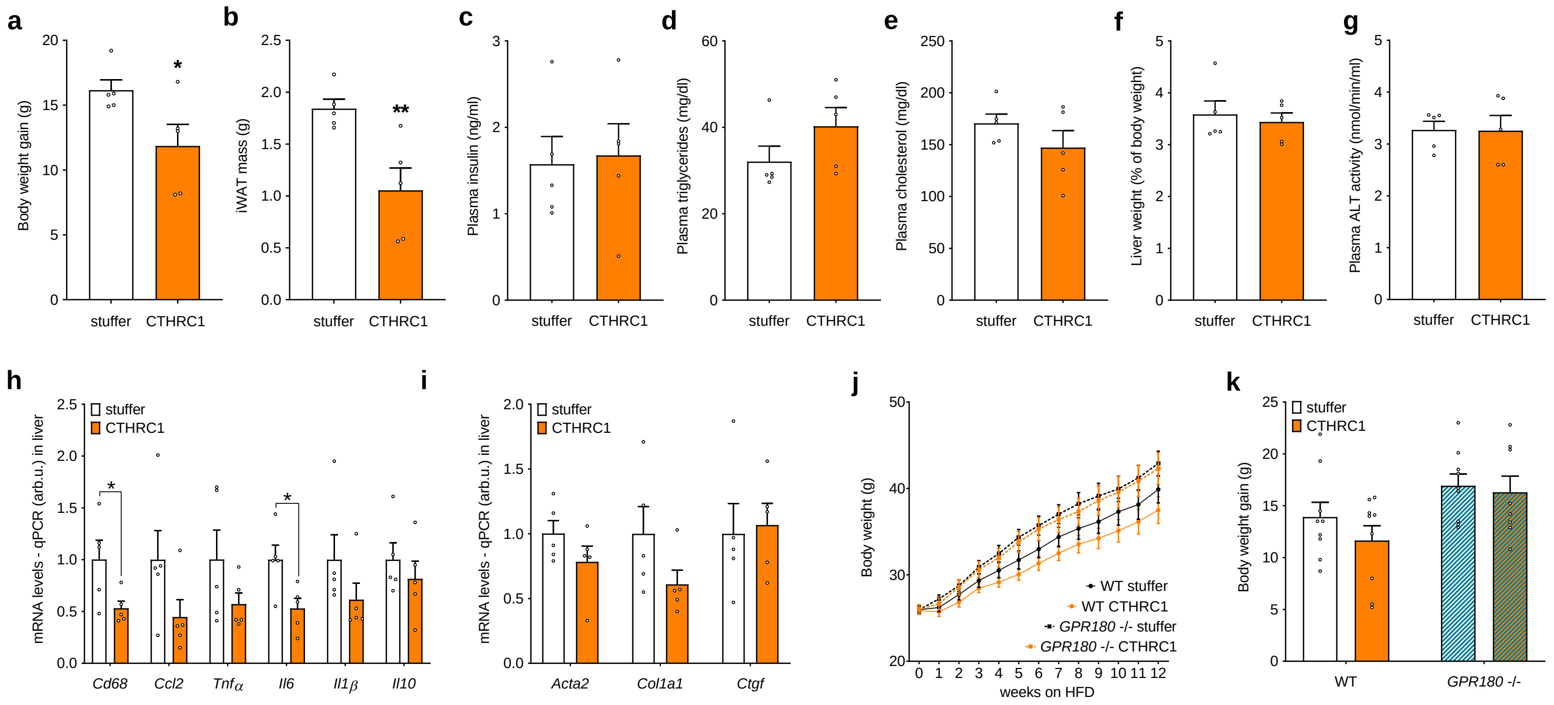

Supplementary Fig. 6 (related to Fig. 6): CTHRC1 ameliorates metabolic disturbances in obesity through GPR180

(a) Body weight gain (p=0.0472) and (b) iWAT mass (p=0.0096) in mice overexpressing CTHRC1 under HFD feeding regimen (n=5). Circulating levels of (c) insulin (p=0.8390), (d) triglycerides (p=0.1829) and (e) cholesterol (p=.0.2453) following AAV-mediated CTHRC1 overexpression in male C57BL/6N mice (n=5). Effect of CTHRC1 overexpression on (f) liver weight (p=0.6503), (g) plasma ALT activity (p=0.9678) and the expression of (h) inflammatory (p=0.0443 for *Cd68* and p=0.0280 for *Il6*) and (i) fibrotic markers (p=0.2029 for *Acta2*; p=0.1378 for *Col1a1* and p=0.8185 for *Ctgf*) in livers of mice fed with HFD (n=5). (j) Body weight (p=0.0616 for treatment) and (k) body weight gain (p=0.3022 for treatment) in wild type and global *GPR180* knockout mice on HFD with stuffer or CTHRC1 overexpression (n=9 for all experimental groups except GPR180-/- CTHRC1 with n=8). Data are shown as mean ± SEM. Statistical significance was calculated using two-sided Student´s t test (Fig. a-i) or two-way ANOVA with repeated measurement (Fig. j and k) and Sidak post-hoc test. Significant differences are indicated as * p < 0.05 and ** p < 0.01. ACTA2, Alpha smooth muscle actin; ALT, Alanine aminotransferase; CCL2, monocyte chemoattractant protein-1; CD68, Cd68 antigen; COL1A1, Collagen type 1 alpha 1; CTGF, Connective tissue growth factor; CTHRC1, Collagen triple helix repeat containing 1; GPR180, G protein coupled receptor 180; HFD, high fat diet; IL1β, Interleukin 1β; IL10, Interleukin 10; IL6, Interleukin 6; iWAT, inguinal white adipose tissue; TNFα, Tumor necrosis factor α; WT, wild type.

Supplementary Table 1 (related to Figure 7): Metabolic profile of individuals with detectable circulating CTHRC1.

parameter	group with detectable circulating CTHRC1	group with circulating CTHRC1 below the limit of detection	p value
age (y)	37.7 ± 1.6	44.8 ± 1.3	0.0008
BMI (kg/m^2)	26.7 ± 0.7	29.4 ± 0.6	0.0028
waist circumference (cm)	98.1 ± 2.1	103.6 ± 1.7	0.0452
fat (%)	23.6 ± 1.0	27.9 ± 0.9	0.0019
adipocyte diameter (µm)	103.9 ± 2.7	116.9 ± 2.3	0.0004
hepatic lipids	5.6 ± 2.5	12.4 ± 2.1	0.0388
extramyocellular lipids	776.0 ± 99.0	1393.3 ± 152.7	0.0010
TAG (mmol/l)	1.3 ± 0.2	1.9 ± 0.1	0.0022
HDL cholesterol (mmol/l)	1.37 ± 0.04	1.27 ± 0.03	0.0300
fasting insulin (mIU/l)	7.44 ± 1.12	10.52 ± 0.93	0.0400
M value (mg/kg BW/min)	6.4 ± 0.4	4.5 ± 0.3	0.0007

Supplementary Table 2: Sequence of siRNAs used in the functional studies (siRNA pools) and secretome screening (single siRNA)

Gene symbol	Species	Sense siRNA sequence
GPR180	Human	#1 CCAGAUUCCGUCUCCACAA<br>#2 GCUGGUUCAGCUUUAGCUA<br>#3 GCGACUACCAAAGAGACAA
CTHRC1	Human	#1 GCUGUCAGCGUUGGUAUUU<br>#2 CCCAACUACAAGCAGUGUU<br>#3 GGAUGUUGCUAUCUGGGUU
TGFβR1	Human	#1 GAACAGAAGUUAAGGCCAA<br>#2 CCAUCUUCACAUGGAGAUU<br>#3 CGAGAUAGGCCGUUUGUAU
TGFβR2	Human	#1 GCAAGACGCGGAAGCUCAU<br>#2 GCUCUGGUGCUCUGGGAAA<br>#3 GAACAUAACACUAGAGACA
SMAD2	Human	#1 GCAGAACTATCTCCTACTA<br>#2 GGAGAAACCTTCCATGCAT<br>#3 GCCACGGTAGAAATGACAA
SMAD3	Human	#1 GCGTGAATCCCTACCACTA<br>#2 GCCATCCATGACTGTGGAT<br>#3 CCGCATGAGCTTCGTCAAA
Gpr180	Mouse	#1 CCAUGCACACCAUCUUAAA<br>#2 GCUAGGAGUGCCUCUCAUA<br>#3 GCUCUCCUCAGUAACGCUA
Non-targeting control siRNA pool	-	#1 UGGUUUACAUGUCGACUAA<br>#2 UGGUUUACAUGUUUUCUGA<br>#3 UGGUUUACAUGUUUUCCUA
ADIPOQ	Human	GAUUGGAGACUUACGUUACUA
ADM	Human	UCGCGUCGGAGUUUCGAAA
AZGP1	Human	CCAGGGAGGACAUCUUUAU
BTD	Human	GCAUGCCUAGUAGCUGUUU
CALU	Human	UCACUGUGGAUGAGCUCAA
CCDC80	Human	GCCUCAUCCCUAAGCUGAU
CHI3L2	Human	GGAGGGUACCUGUUUGGUU
CTHRC1	Human	GCUGUCAGCGUUGGUAUUU
ECM2	Human	GCAGAGGAGGAGACAGAAA
EFEMP1	Human	CCACCAAAGAUGCGUGAAU
ENPP2	Human	CCAAUUAUCCAGGGAUUAU
FBLN2	Human	GUCGUGUGAGUCCAAUCCUAA
FSTL1	Human	GCAGUAAUGGCAAGACCUA
HP	Human	GGUUCGCUACCAGUGUAAGAA
IGFBP3	Human	CCAGCUCCAGGAAAUGCUA
IGFBP7	Human	CCUCAUCUGGAACAAGGUA
INHBA	Human	CCGAGGAAGUGGGCUUAAA

ISLR	Human	CCCUCCAAUUGCUCAAGAU
ITIH1	Human	GCCGUUACAGCAGAUGGAA
ITIH5	Human	GCAAGUACGAGCACAGCAU
LGALS3BP	Human	GCGUGAACGAUGGUGACAU
LTBP2	Human	GCACCAACCACUGUAUCAA
MFGE8	Human	CCUGGAGAAUGGGAACAUU
PCOLCE	Human	GCACCUUGCAGAGCAACUU
PCOLCE2	Human	GGAUGUGUACAAUGGCCAU
PLTP	Human	GCAAAGAAGGCCACUUCUA
PODN	Human	GCUCAGCCACACACAACUA
PTGDS	Human	GGGCUGAGUUAAAGGAGAA
RBP4	Human	CCAGACUCUGAUUCAUUAA
SEMA3G	Human	CCGUCUGUGUGUACCACAU
SERPINI1	Human	GCUGACUUGUCAGUGAAUA
SERPINE2	Human	GCCTCTTTCCGGCTGTGAC
SERPINF1	Human	GCGAACAGAAUCCAUCAUU
SPON2	Human	UCUCGUUUGUGGUGCGCAU
SVEP1	Human	GCACAUGUGUGAAAGGAUU
TF	Human	GGUUUGGUGUAUGAUGCUU

Supplementary Table 3: Oligo duplexes targeting human *GPR180* gene used for generation HEK293T stable cell line with receptor deletion.

duplex	Oligonucleotide sequence
hGPR180gRNA1	FWD: CACCGactcgaagtggccgatgcgc<br>REV: AAACgcgcatcggccacttcgagtC
hGPR180gRNA2	FWD: CACCGtgccccgcagggtcttaccc<br>REV: AAACgggtaagaccctgcggggcaC
hGPR180gRNA3	FWD: CACCGcggccaccagcagcacgtga<br>REV: AAACtcacgtgctgctggtggccgC
hGPR180gRNA4	FWD: CACCGgacgcccagggccagcgcat<br>REV: AAACatgcgctggccctgggcgtcC
hGPR180gRNA5	FWD: CACCGctgtggccctcacgtgctgc<br>REV: AAACgcagcacgtgagggccacagC
hGPR180gRNA6	FWD: CACCGacgtgctgctggtggccgca<br>REV: AAACtgcggccaccagcagcacgtC

Supplementary Table 4: Sequence of qPCR primers used in the study.

Gene symbol	Species	Primer sequence
ACSL5	Human	FWD: AAGAAGGACAGGGACTCGTG<br>REV: AGCAGCAGGAAATTCAGACC
ADIPOQ	Human	FWD: GGTGAGAAGGGTGAGAAAGGA<br>REV: TTTCACCGATGTCTCCCTTAG
B2M	Human	FWD: CGCTCCGTGGCCTTAGC<br>REV: AATCTTTGGAGTACGCTGGATAGC
CIDEA	Human	FWD: GGCAGGTTCACGTGTGGATA<br>REV: GAAACACAGTGTTTGGCTCAAGA
COX7A1	Human	FWD: TGACATCCCGTTGTACCTGAAG<br>REV: ACAGTGCCGCCCAGACA
CPT1B	Human	FWD: AAACAGTGCCAGGCGGTC<br>REV: CGTCTGCCAACGCCTTG
DIO2	Human	FWD: TCGATGCCTACAAACAGGTG<br>REV: TGGCTCCCTCAGCTATCTTC
ELOVL3	Human	FWD: AACCTGCAAGGGCCTCTC<br>REV: ATAATGCCCCACATCCTCAC
FABP4	Human	FWD: AACCTTAGATGGGGGTGTCCTGGT<br>REV: ACGCCTTTCATGACGCATTCCACC
GPR180	Human	FWD: CCAGATGCCGAAGGGAAT<br>REV: GGAGGAAAAAGAACTCATGTAACC
PGC1A	Human	FWD: CAGCCTCTTTGCCCAGATCTT<br>REV: TCACTGCACCACTTGAGTCCAC
PPARG2	Human	FWD: CGTGGCCGCAGAAATGA<br>REV: TCAAAGGAGTGGGAGTGGTC
RPL13A	Human	FWD: GGACCGTGCGAGGTATGCT<br>REV: ATGCCGTCAAACACCTTGAG A
UCP1	Human	FWD: AGGTCCAAGGTGAATGCCC<br>REV: GCGGTGATTGTTCCCAGGA
Acsl5	Mouse	FWD: ACAACCAGTCTGTGGGGATT<br>REV: GGTCATTGTTCTTCTGGAAAGC
Acta2	Mouse	FWD: ACTGGGACGACATGGAAAAG<br>REV: GTTCAGTGGTGCCTCTGTCA
Adipoq	Mouse	FWD: ACCATGCTCTACGTGCTGTG<br>REV: CGATTGTCAGTGGATCTGACG
Ccl2	Mouse	FWD: CATCCACGTGTTGGCTCA<br>REV: GATCATCTTGCTGGTGAATGAGT
Cd68	Mouse	FWD: CACAGTGGACATTCATGGCG<br>REV: GCAAGAGAAACATGGCCCGA
Cidea	Mouse	FWD: ACTTCCTCGGCTGTCTCAATGTCA<br>REV: TCAGCAGATTCCTTAACACGGCCT
Cpt1b	Mouse	FWD: AGTCATGGTGGGCAACTAAC<br>REV: TGCTTGCACATTTGTGTTCTT
Col1a1	Mouse	FWD: CATGTTCAGCTTTGTGGACCT<br>REV: GCAGCTGACTTCAGGGATGT
Cox7a1	Mouse	FWD: CAGCGTCATGGTCAGTCTGT<br>REV: AGAAAACCGTGTGGCAGAGA

Ctgf	Mouse	FWD: TGACCTGGAGGAAAACATTAAGA<br>REV: AGCCCTGTATGTCTTCACACTG
Dio2	Mouse	FWD: GAACCATGAAGCCAACGACT<br>REV: CTTCCTCCTAGATGCCTACAAAC
Elovl3	Mouse	FWD: GGCATAATTGTTACCTGATTCAGG<br>REV: GATGGTTCTGGGCACCATCTT
Fabp4	Mouse	FWD: GATGCCTTTGTGGGAACCT<br>REV: CAACAGTAGCATCCTGAGCCCT
Gpr180	Mouse	FWD: CAGAGATGGGCTAGGAGTGC<br>REV: GCCCATACACAGGCTCAGAA
Il1b	Mouse	FWD: AGTTGACGGACCCCAAAAG<br>REV: AGCTGGATGCTCTCATCAGG
Il10	Mouse	FWD: GTAGAAGTGATGCCCCAGGC<br>REV: AAATCGATGACAGCGCCTCAG
Il6	Mouse	FWD: GCTACCAAACTGGATATAATCAGGA<br>REV: CCAGGTAGCTATGGTACTCCAGAA
Pgc1a	Mouse	FWD: ACCCCAAAGGATGCGCTCTCGTT<br>REV: TGCGGTGTCTGTAGTGGCTTGATT
Pparg2	Mouse	FWD: GCATGGTGCCTTCGCTGA<br>REV: TGGCATCTCTGTGTCAACCATG
Tbp	Mouse	FWD: GAAGCTGCGGTACAATTCCAG<br>REV: CCCCTTGTACCCTTCACCAAT
Tnfa	Mouse	FWD: CAGGGGCCACCACGCTCTTC<br>REV: TACGACGTGGGCTACAGGCTTGTC
Ucp1	Mouse	FWD: GGGCATTCAGAGGCAAATCAGCTT<br>REV: ACACTGCCACACCTCCAGTCATTA
