# Supplementary material for: GPR180 is a component of TGFβ signalling that promotes thermogenic adipocyte function and mediates the metabolic effects of the adipocyte-secreted factor CTHRC1
Source: Nat Commun. 2021 Dec 8;12:7144. doi: 10.1038/s41467-021-27442-x (PMC8655035; doi:10.1038/s41467-021-27442-x)
Supplement: Supplementary file 3 — Description of Additional Supplementary Files [file 41467_2021_27442_MOESM3_ESM.pdf]

Description of Additional Supplementary Files

File Name: Supplementary Data 1
Description: The overlap of differentially expressed genes between human brown vs white adipose tissue and brown vs white hMADS cells.

File Name: Supplementary Data 2
Description: Pathway analysis of DEGs obtained by RNA-seq of brown hMADS cells following GPR180 silencing.

File Name: Supplementary Data 3
Description: Proteomic analysis of cell-conditioned media using liquid chromatography coupled to tandem mass spectrometry

File Name: Supplementary Data 4
Description: Genes that are significantly expressed in human mature adipocytes and encode for a secreted protein evidenced by the presence of signal peptide sequence.
