# Supplementary material for: GPR180 is a component of TGFβ signalling that promotes thermogenic adipocyte function and mediates the metabolic effects of the adipocyte-secreted factor CTHRC1
Source: Nat Commun. 2021 Dec 8;12:7144. doi: 10.1038/s41467-021-27442-x (PMC8655035; doi:10.1038/s41467-021-27442-x)
Supplement: Supplementary file 8 — Reporting Summary [file 41467_2021_27442_MOESM8_ESM.pdf]

nature portfolio

Corresponding author(s): Christian Wolfrum

Last updated by author(s): 15.11.2021

Reporting Summary

Nature Portfolio wishes to improve the reproducibility of the work that we publish. This form provides structure for consistency and transparency in reporting. For further information on Nature Portfolio policies, see our Editorial Policies and the Editorial Policy Checklist.

Statistics

For all statistical analyses, confirm that the following items are present in the figure legend, table legend, main text, or Methods section.

n/a	Confirmed	
☐	☒	The exact sample size (*n*) for each experimental group/condition, given as a discrete number and unit of measurement
☐	☒	A statement on whether measurements were taken from distinct samples or whether the same sample was measured repeatedly
☐	☒	The statistical test(s) used AND whether they are one- or two-sided<br>*Only common tests should be described solely by name; describe more complex techniques in the Methods section.*
☒	☐	A description of all covariates tested
☐	☒	A description of any assumptions or corrections, such as tests of normality and adjustment for multiple comparisons
☐	☒	A full description of the statistical parameters including central tendency (e.g. means) or other basic estimates (e.g. regression coefficient) AND variation (e.g. standard deviation) or associated estimates of uncertainty (e.g. confidence intervals)
☐	☒	For null hypothesis testing, the test statistic (e.g. *F*, *t*, *r*) with confidence intervals, effect sizes, degrees of freedom and *P* value noted<br>*Give P values as exact values whenever suitable.*
☒	☐	For Bayesian analysis, information on the choice of priors and Markov chain Monte Carlo settings
☒	☐	For hierarchical and complex designs, identification of the appropriate level for tests and full reporting of outcomes
☐	☒	Estimates of effect sizes (e.g. Cohen's *d*, Pearson's *r*), indicating how they were calculated

Our web collection on statistics for biologists contains articles on many of the points above.

Software and code

Policy information about availability of computer code

Data collection	Western blots were visualized by the Image Quant system (GE Healthcare Life Sciences). Quantitative PCR was performed using ViiA7 real-time PCR system (Applied Biosystems). Image acquisition for immunoflorescence was done by Operetta automated microscope (Perkin Elmer). Biochemical colorimetric assays or luminiscence was detected by SynergyMx plate reader (BioTek). Cellular respiration was monitored by XF96 Extracellular Flux Analyzer (Agilent Seahorse). Indirect calorimetry measurement was performed by automated home cage phenotyping Phenomaster (TSE-systems). Live mice body composition was measured with a magnetic resonance imaging technique (EchoMRI 130, Echo Medical Systems). Surface temperature was recorded by infrared camera (E60; FLIR). Tissue section were visualized by Axiophot microscope equipped with AxioCam MR (Zeiss). Cell sorting ws done by Sony SH800S (Sony Biotechnology). Electroporation of gRNA was performed by ECM830 electroporator (BTX Harvard Apparatus).
Data analysis	Quantification of Western blots was done by ImageJ v 1.53e (NIH). Real-time PCR was analyzed by ViiA7 Ruo v1.2.3 (ThermoFisher). Immunofluorescence image analysis was performed either by Harmony v3.5 (Perkin Elmer) or Matlab2019 (MathWorks). Colorimetric assays were analyzed by Gen5 v3.08 (BioTek). Cell respiratory analysis was done by Wave 2.6.0 (Agilent Seahorse). Energy expenditure analysis was performed by Phenomaster software v5.6.5 (TSE-systems). Fat and lean mass was analyzed using Echo MRI 14 software. Surface temperature was analyzed by FLIR Tools software (FLIR). Statistical analysis was performed by GraphPad Prism 8.

For manuscripts utilizing custom algorithms or software that are central to the research but not yet described in published literature, software must be made available to editors and reviewers. We strongly encourage code deposition in a community repository (e.g. GitHub). See the Nature Portfolio guidelines for submitting code & software for further information.

Data

Policy information about availability of data

All manuscripts must include a data availability statement. This statement should provide the following information, where applicable:

- Accession codes, unique identifiers, or web links for publicly available datasets
- A description of any restrictions on data availability
- For clinical datasets or third party data, please ensure that the statement adheres to our policy

The RNA sequencing data of hMADS cells after ablation of GPR180 denerated in this study have been deposited in The European Nucleotide Archive under accession number PRJEB38756 (https://www.ebi.ac.uk/ena/browser/view/PRJEB38756?show=reads). The RNA sequencing data from the clinical transcriptome study used in this study are available in The European Nucleotide Archive, accession number PRJEB23275 (https://www.ebi.ac.uk/ena/browser/view/PRJEB23275?show=reads). The mass spectrometry proteomics data have been deposited to the ProteomeXchange Consortium via the PRIDE partner repository with the dataset identifier PXD029335. Supporting data is included in this article as supplementary data 1-4. Source data are provided with this paper.

Field-specific reporting

Please select the one below that is the best fit for your research. If you are not sure, read the appropriate sections before making your selection.

☒ Life sciences ☐ Behavioural & social sciences ☐ Ecological, evolutionary & environmental sciences

For a reference copy of the document with all sections, see nature.com/documents/nr-reporting-summary-flat.pdf

Life sciences study design

All studies must disclose on these points even when the disclosure is negative.

Sample size	Sample size was determined based on previous experiments in our lab and similar studies reported in the literature. (Balaz et al. 2019 Cell Metab; Sun et al. 2018 Nat Med; Sun et al. 2020 Nature)
Data exclusions	In cell culture and animal experiments, no samples and individuals were excluded from analyses. In RNA-seq analysis of human supraclavicular brown adipose tissue and subcutaneous white adipose tissues, one outlier samples was removed based on the PCA plots after vst normalization of top 500 most variable genes.
Replication	Unless stated otherwise, all cell culture experiments were independently reproduced 2-4 times. All animal experiments were repeated independently 2 or 3 times.
Randomization	For each study, all animals (littermates) were randomly allocated into experimental groups. Regarding cell culture experiments, culture wells were randomly assigned to treatments in each independent round of experiment to avoid any plate effect.
Blinding	If not stated otherwise, investigators were blinded to allocation of mice to sample groups during experiments and data analysis. Metabolic phenotyping of mice was performed by technicians unaware of the study.

Reporting for specific materials, systems and methods

We require information from authors about some types of materials, experimental systems and methods used in many studies. Here, indicate whether each material, system or method listed is relevant to your study. If you are not sure if a list item applies to your research, read the appropriate section before selecting a response.

Materials & experimental systems

n/a	Involved in the study	
☐	☒	Antibodies
☐	☒	Eukaryotic cell lines
☒	☐	Palaeontology and archaeology
☐	☒	Animals and other organisms
☐	☒	Human research participants
☒	☐	Clinical data
☒	☐	Dual use research of concern

Methods

n/a	Involved in the study	
☒	☐	ChIP-seq
☒	☐	Flow cytometry
☒	☐	MRI-based neuroimaging

Antibodies

Antibodies used	UCP1 (ThermoFisher, Cat# PA1-24894, RRID: AB_2241459), γ-TUBULIN (Sigma-Aldrich, Cat# T-5326, RRID: AB_532292), RFP (Evrogen, Cat# AB233, RRID:AB_2571743), HSP90 (Cell Signaling, Cat# 4877, RRID: AB_2233307), phospho-SMAD3 Ser423/425 (Abcam, Cat# ab52903, RRID:AB_882596), phospho-SMAD3 Ser423/425 (Cell Signaling, Cat# 9520, RRID:AB_2193207), SMAD3 (Abcam, Cat# ab28379, RRID:AB_2192903), SMAD3 (Cell Signaling, Cat# 9523, RRID:AB_2193182), HA (Cell Signaling, Cat# 3724, RRID:AB_1549585), V5 (Invitrogen, Cat# R960-25, RRID:AB_2556564), phospho-HSL Ser660 (Cell Signaling, Cat# 4126, RRID: AB_490997), TGFβ 1,2,3 (R&D Systems, Cat# MAB1835, RRID:AB_357931), CTHRC1 (Sigma Aldrich, Cat# SAB2107469, RRID:AB_2819006), Oxphos (Abcam, Cat# ab110413, RRID:AB_2629281), phospho-p44/42 MAPK (ERK1/2) Thr202/Tyr204 (Cell Signaling, Cat# 4370, RRID:AB_2315112), p44/42 MAPK (ERK1/2) (Cell Signaling, Cat# 4695, RRID:AB_390779), phospho-p38 MAPK Thr180/Tyr182 (Cell Signaling, Cat# 4511, RRID:AB_2139682), p38 MAPK (Cell Signaling, Cat# 8690, RRID:AB_10999090), TGFβR1 (Abcam, Cat# ab31013, RRID:AB_778352), TGFβR2 (Abcam, Cat# ab186838, RRID:AB_2728775), phospho-CREB Ser133 (Cell Signaling, Cat# 9198, RRID: AB_2561044), phospho-PKA substrates (Cell Signaling, Cat# 9624, RRID:AB_331817), phospho-AKT Thr308 (Cell Signaling, Cat# 13038, RRID: AB_2629447), phospho-AMPK Thr172 (Cell Signaling, Cat# 2535, RRID:AB_331250), phospho-FAK Tyr397 (Cell Signaling, Cat# 2599, RRID:AB_2106814), phospho-Foxo1 Thr24 (Cell Signaling, Cat# 8556, RRID:AB_10891442), phospho-JNK Thr183 (Cell Signaling, Cat# 9251, RRID:AB_331659), phospho-SMAD1/5/9 Ser463/465 (Cell Signaling, Cat# 13820, RRID:AB_2493181), anti-mouse HRP secondary (Merck, Cat# 401253, RRID: AB_437779), anti-rabbit HRP secondary (Merck, Cat# 401393, RRID: AB_10683386), anti-mouse Alexa Fluor 488 secondary (Thermo Fisher, Cat# A-11029, RRID:AB_2534088), anti-rabbit Alexa Fluor 488 secondary (Thermo Fisher, Cat# A-21206, RRID:AB_2535792)
Validation	Primary antibodies used in this study were validated either by siRNA mediated knockdown of target proteins (CTHRC1, TGFβR1 and 2, SMAD3), overexpression (RFP and epitope tags V5 and HA) or with the use of positive controls (TGFβ treatment for validation of phospho-SMAD3 Ser423/425, recombinant TGFβ for validation of TGFβ 1,2,3 antibody and forskolin treatment for validation of phospho-CREB Ser133 and phospho-PKA substrates antibodies).<br>Other antibodies such as phospho-HSL Ser660 (isoproterenol), phospho-SMAD1/5/9 Ser463/465 (BMP4), phospho-AKT Thr308 and phospho-Foxo1 Thr24 (insulin) were previously validated in laboratory in other experiments including positive control (validation method indicated in brackets). In addition, several widely used and accepted antibodies, which were validated by manufacturers and other research groups were used (UCP1, γ-tubulin, HSP90, oxphos, phospho-AMPK Thr172, phospho-p38 MAPK Thr180/Tyr182, phospho-p44/42 MAPK (ERK1/2) Thr202/Tyr204, phospho-JNK Thr183 and phospho-FAK Tyr397).

Eukaryotic cell lines

Policy information about cell lines

Cell line source(s)	hMADS cells - Dr. Ez-Zoubir Amri, University of Nice, France (Elabd et al., 2009)<br>murine immortalized brown adipocytes (iBAs) - prof. Ronald C. Kahn, Harvard University, Boston, USA (Klein et al., 2002)<br>HEK293T (ThermoFisher)<br>HEK293LTV (Cell Biolabs)
Authentication	hMADS and iBA cell lines are commonly used to study brown adipocyte physiology and function, and were extensively tested in our previous studies as well as by other groups. These cell lines were authenicated by PCR assay using species-specific primers to determine expression level of key thermogenic protein UCP1. HEK293T cell line is widely used in cell biology research for reporter assays or signalling studies. HEK293LTV cell line is derived from HEK293T and used for higher lentiviral yields. None of HEK293 cell lines was authenticated.
Mycoplasma contamination	All cell lines used in the study were repeatedly tested negative for mycoplasma contamination every three months.
Commonly misidentified lines<br>(See ICLAC register)	No commonly misidentified cell lines were used.

Animals and other organisms

Policy information about studies involving animals; ARRIVE guidelines recommended for reporting animal research

Laboratory animals	Mus musculus, strains C57BL6/N (Charles River), GPR180 null mice, GPR180 floxed, GPR180 floxed x Adiponectin CreERT2 mice, all adult males (8-20 weeks). For more details, please see methods section or figure legends.
Wild animals	Study did not involve wild animals.
Field-collected samples	Study did not involve samples collected from the field.
Ethics oversight	All animal procedures were approved by the Veterinary office of the Canton of Zürich. Health status of all mouse lines was regularly monitored according to FELASA guidelines.

Note that full information on the approval of the study protocol must also be provided in the manuscript.

Human research participants

Policy information about studies involving human research participants

Population characteristics	For detailed characteristic of population, please see Orava et al., 2011 Cell Metab (The clinical transcriptome study) and

Population characteristics	Balaz et al., 2014 Obesity (The clinical study with normal weight participants and participants with obesity and differing glycaemic control).
Recruitment	The clinical transcriptome study - The subjects were screened for medical history and status, and only healthy volunteers were enrolled in the study.<br>The clinical study with normal weight participants and participants with obesity and differing glycaemic control - Eighty-five middle-aged sedentary men were recruited, while patients with chronic disease or regular use of pharmacotherapy were excluded.<br>All study participants provided witnessed written informed consent prior entering the study. For more details please see Methods section of the article.
Ethics oversight	The clinical transcriptome study was approved by the Ethics Committee of the Hospital District of Southwest Finland and conducted according to the principles of the Declaration of Helsinki. The clinical study with normal weight participants and participants with obesity and differing glycaemic controlwas approved by the Local Ethics Committee of the University Hospital in Bratislava, Slovakia and it conforms to the ethical guidelines of the 2000 Helsinki declaration.

Note that full information on the approval of the study protocol must also be provided in the manuscript.
